# Evaluation of the effect of dextrose prolotherapy versus deep dry needling therapy for the treatment of temporomandibular joint anterior disc displacement with reduction: (a randomized controlled trial)

**DOI:** 10.1007/s00784-024-05830-z

**Published:** 2024-08-08

**Authors:** Amr Gibaly, Mohamed Abdelmoiz, Ahmed Nagi Alghandour

**Affiliations:** Department of Oral and Maxillofacial Surgery, Faculty of Dental Medicine, Beni-Suef University, East Nile educational compound, Beni-Suef, 62764 Egypt

**Keywords:** Dextrose prolotherapy injection, Deep dry needling, Muscular trigger points, Intraarticular injection, And TMJ anterior disc displacement

## Abstract

**Objective:**

to compare the combined effect of Prolotherapy and Deep Dry Needling (DDN) versus DDN effect on relieving the symptoms of Temporomandibular joint (TMJ) anterior disc displacement.

**Patients and methods:**

The clinical trial randomly allocated forty patients. The ***(control group)*** patients received four intraarticular and masseteric DDN sessions, while the ***(study group)*** patients were subjected to the exact technique followed by Prolosolution injection. The baseline preoperative measurements included Maximal interincisal opening (MIO), auscultation of the presence of clicking, and Visual Analogue Scale (VAS), which were repeated for postoperative measurements after one, two, five, and eight months.

**Results:**

By the end of the study, all patients expressed apparent improvement in pain MIO and clicking. The inter- and intragroup comparison revealed that the pain score values of the control group after five and eight months were significantly higher than those of the study group. The study group demonstrated more significant MIO calibration than the control group, with insignificant differences between both groups regarding the presence of clicking at any time interval. The associations between clicking and VAS values, between clicking and MIO, and between VAS values and increased MIO were positive in the test group and negative in the control group.

**Conclusions:**

Dextrose Prolotherapy and DDN were beneficial. However, Prolotherapy demonstrated more significant, sustained, and correlated long-term alleviation of symptoms and increased MIO.

**Clinical relevance:**

The study assesses the sole effect of dextrose prolotherapy on relieving the signs of TMJ anterior disc displacement apart from the impact of the penetrating needle.

**Clinical trial registration:**

The study was registered on www.clinicaltrials.gov (#: NCT05821985) by Ahmed Nagi Alghandour.

**Supplementary Information:**

The online version contains supplementary material available at 10.1007/s00784-024-05830-z.

## Introduction

The TMJ represents a complex synovial articulation, with its articular disc enclosed between the articular tubercle’s slope and the convexity of the mandibular condyle [1]. Although the magnetic resonance imaging (MRI) interpretation of the normal discal position defines the posterior discal band atop the highest convexity of the mandibular condyle, at a 12 o’clock position, this discal disposal is altered in almost 30% of the population without physical signs. This means the treatment of discal displacement mainly depends on alleviating the signs and symptoms rather than restoring the anatomical disc position [2, 3].

Various non-invasive therapeutic modalities are utilized for relieving the signs and symptoms of anterior discal displacement, including physical therapy, exercises, ischemic compression, heat therapy, acupuncture, dry needling, wet needling injections with different agents, and pharmacological treatments [4].

Among those, the trigger point injection is an effective modality, with or without the infusion of saline or ringer’s solutions, hyaluronic acid, corticosteroids, local anesthetics, botulinum toxin, platelet-rich plasma, or hypertonic dextrose [5].

The Hypertonic dextrose injection prolotherapy is inexpensive, readily available, and safe proliferative injection therapy that aims to trigger a low-grade inflammatory response inside the TMJ, with the resultant captivation of abundant fibroblasts that regenerate and strengthen the tendinous and ligamentous attachments and stabilize the disc and the fibro-osseous junctions [6].

On the other hand, dry needling refers to inserting needles without an injectate. Dry needling is beneficial for treating a variety of neuromusculoskeletal pain syndromes as it represents a treatment modality for the ligaments and tendons, muscles, subcutaneous fascia, peripheral nerves, and neurovascular bundles [7, 8]. Deep dry needling is a technique that utilizes the intracapsular insertion of dry needles to approach the discal insertion to the lateral pterygoid muscle and the masseteric muscle origin, along the zygomatic bone and arch, aiming to inactivate the muscular trigger points (TPs) [9].

The current study represents a randomized controlled clinical trial that aims to assess the sole effect of the dextrose prolotherapy solution on relieving the signs of TMJ anterior disc displacement apart from the impact of the penetrating needle, by injecting 12.5% of dextrose solution intraarticular and into the myofascial trigger points versus the DDN utilizing the exact needle and needling technique of the same anatomic sites at the same time intervals. Meanwhile, the authors are evaluating pain reduction as a primary outcome and the MIO and clicking persistence as predictor variables, as we hypothesize that the null hypothesis is that there is no difference between both tested groups in pain, clicking, and maximum mouth opening.

## Materials and methods

### Study design

The clinical trial’s population consisted of all patients presenting to the outpatient clinics of Shiek-Zayed Cairo public hospital, the Faculty of Dental Medicine of Beni-Suef University, and the Faculty of Dentistry of Modern University for technology & information and diagnosed with bilateral TMJ anterior disc displacement with reduction between April 2023 and December 2023.

#### Inclusion criteria

1. Patients of both sexes with an age range of 18–45 years, with the clinical signs of pain, limitation of mouth opening, and TMJ clicking.
2. The (MRI) interpretation of bilateral TMJ disc displacement with reduction; Wilkes classification type II.
3. With an associated masseteric TP along its attachment to the zygomatic arch.

#### Exclusion criteria

1. The clinical or radiographic signs of disc displacement without reduction, osteoarthritis, or articular bony changes.
2. Previous TMJ surgery, arthrocentesis, or occlusal splints, or the presence of any systemic disease that would affect the TMJ anatomy or mechanical function.
3. A history of allergic reactions to any components of the injectable solution.

The study represents a randomized controlled clinical trial registered on www.clinicaltrials.gov (Registration #: NCT05821985) and approved by the research ethics committee of the faculty of Dentistry, Beni-Suef University (Approval #: REC-FDBSU/06042023-05/GA). It followed the “Helsinki Declaration for the ethical principles for medical research involving human subjects.”

### Randomization and allocation concealment

An independent staff member equally distributed the patients into study and control groups, labeling them in sequential numbers and randomly allocating them, aided by the Microsoft Excel spreadsheet. The assigned randomization was concealed in sealed opaque envelopes and provided to the operator before the first treatment session.

### Preoperative measurements

All patients within both groups were subjected to baseline preoperative measurements that included:

1. The individual patient’s assessment of his pain and tenderness upon manual palpation through VAS. From (0–10) grades, zero denotes the absence of pain, while ten represents the worst pain the patient has ever had.
2. A record of an unassisted maximal interincisal opening MIO in millimeters. The patients opened their mouths widely while sitting upright, and the distance between the incisal edges of the upper and lower central incisors was calibrated by a ruler.
3. The auscultation of TMJ clicking at the preauricular region.

The authors considered VAS pain reduction the primary outcome, as pain upon mouth opening represented the patient’s chief complaint. On the other hand, the increase in MIO and the alleviation of clicking were considered secondary outcomes.

### Operative procedure

The patients within the (control group) received four sessions of intraarticular and masseteric DDN insertion in a fast in and fast out technique aided by a 25 gauge needle and 3.8 cm length with a time interval of 15 days between each session, following a timeline of (baseline, two weeks, four weeks, and six weeks). On the other hand, the patients within the (study group) were subjected to the exact technique on the same anatomical points at the same time intervals, followed by intraarticular and masseteric TPs infusion of (12.5% Dextrose Prolotherapy solution).

After positioning the patient supine and swabbing the skin overlying the target area with povidone-iodine (betadine), the mandibular condyle and the target areas were palpated and marked with a washable felt-tip pen.

#### Point identification

**Fig. 1:**
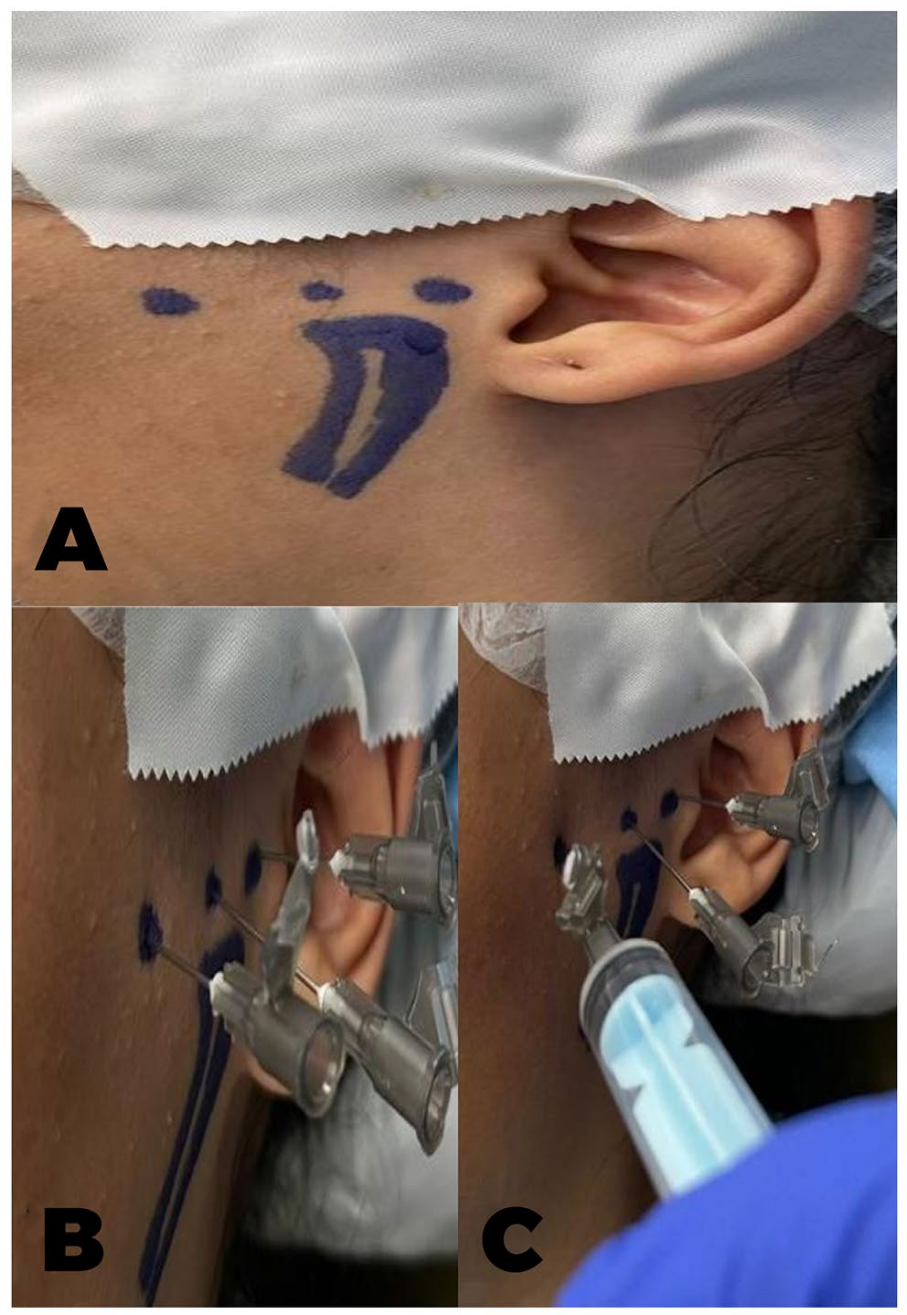
**A**) The tracing and labeling of the mandibular condyle and the three anatomical landmarks for needle insertion. **B**) The needles are inserted into the demarcated insertion points among both groups. **C**) The deposition of the Dextrose Prolotherapy solution among the test group patients

The mandibular condyle and three anatomical needle insertion points were traced and marked for every patient bilaterally. The first anatomic point represented a point of needle insertion into the posterior condylar aspect, which is palpated as the bony depression formed anterior to the tragus by the forward and downward condylar translation after utilizing a bite block for maximal mouth opening. The second anatomical point represented the anterior discal attachment to the upper head of the lateral pterygoid muscle, which was palpated as the depression located just anterior to the mandibular condyle upon mouth closure after the removal of the bite block. The third point represented the trigger point along the masseteric muscle attachment to the inferior border of the zygomatic arch after asking the patients to clench their teeth, which was confirmed to be the most tender and rigid spot to palpation. (Figure 1A)

#### Deep dry needling technique (control group)

After the target points were identified, they were needled according to Hong [10] forward and backward in a fast in and fast out technique without depositing an injectate using hypodermic needles of 25 gauge and 3.8 cm length, inserted through the skin to an approximate depth of one centimeter, and held in place for 20 s before they were removed to simulate the time needed for the deposition of the injectate in the test group. The first posterior condylar needle insertion utilized maximal mouth opening aided by a bite block that kept the mouth open during the procedure. The needle was directed medially and anteriorly to approach the medial aspect of the glenoid fossa and avoid approaching the external auditory canal. The second anterior condylar needle was inserted while the mouth was closed in a posteromedial direction to approach the discal insertion to the upper head of the lateral pterygoid muscle. The third masseteric TP injection was undertaken while the patients clenched their teeth. It was directed at an acute angle of (30^O^) after being warned that the insertion would be painful and wound elect muscle twitch or radiating pain. Then, the patient’s head was turned, and the same procedure was repeated on the other side. (Figure 1B)

#### The prolotherapy injection technique (study group)

The dextrose prolotherapy solution was prepared by diluting (1.5 ml.) of 25% Dextrose into a concentration of 12.5% by mixing it with (0.75 ml. Saline solution and 0.75 ml. Lidocaine). Following the same dry needling technique utilized for the control group, a plastic syringe containing 3 ml. of the prepared solution was connected to the needle to slowly infuse 1 ml of the solution at each target point after aspiration. (Figure 1C)

#### Postoperative care

Among both groups, the minute bleeding was controlled by firm gauze compression over the area of needle punctures for a few minutes. The patients were instructed to avoid using medications throughout treatment, except for Panadol (Panadol; 500 mg tab. GLAXO SMITHKLINE, Bentford, UK) only if needed. On the other hand, advised to follow an open and close mouth exercise three times daily, each count of ten gradual maximal mouth openings throughout the treatment phase, and instructed to follow a regimen of soft diet, avoid biting hard food or objects, and apply hot fomentation five times daily for two days following every treatment session.

### The data collection method

The patients of both groups were recalled for postoperative measurements after one month (after two sessions of treatment), two months (after two weeks of the last treatment sessions), five months (after three months of the last treatment session), and after eight months (after six months of last session). Upon each recall visit, the same preoperative measures of VAS, MIO, and the auscultation of the presence or absence of TMJ clicking were recorded in the same manner.

The preoperative baseline and the four postoperative measurement groups were recorded, tabulated, and submitted for statistical analysis.

### Statistical analysis

The categorical data were presented as frequency and percentage values and analyzed using the chi-square test for intergroup comparisons and Cochran’s Q test, followed by multiple McNemar’s tests with Bonferroni correction for the intragroup comparisons. The numerical data was presented as mean, standard deviation (SD), median, and interquartile range (IQR) values. They were analyzed for normality by checking data distribution and using Shapiro-Wilk’s test. Age and MIO data were normally distributed. They were studied using an independent t-test for intergroup comparisons and repeated measures ANOVA followed by a Bonferroni post hoc test for intragroup comparisons. Pain score data were non-parametric and were analyzed using the Mann-Whitney U test for intergroup comparisons and Friedman’s test, followed by the Nemenyi post hoc test for intragroup comparisons. Correlations were analyzed using Spearman’s rank-order correlation coefficient. The significance level was set at *p* < 0.05 within all tests, with a confidence interval of 95%. The statistical analysis was performed with R statistical analysis software version 4.3.2 for Windows (R Core Team 2023. R Foundation for Statistical Computing, Vienna, Austria.)

## Results

A power analysis was designed to have adequate power to apply a two-sided statistical test of the null hypothesis that there is no difference between both tested groups in pain, clicking, and maximum mouth opening. By adopting (α) and (β) levels of (0.05), the power of 95%, and effect size (d) of (1.38), calculated based on the study published by Refai et al. [11]. The total required sample size (n) was found to be (30) cases. However, it was increased to forty patients to account for the possible dropouts during the follow-up period (i.e., 20 cases per group). The Sample size calculation was performed by (R statistical analysis software, version 4.3.2 for Windows. R Core Team 2023; R Foundation for Statistical Computing, Vienna, Austria.)

Forty patients were assigned to this clinical trial, equally and randomly allocated to the study and control groups: twenty patients each. The study group included eight males and twelve females, versus seven males and thirteen females for the control group. The mean age of patients in the study group was recorded (31.70 ± 7.81) years versus (31.85 ± 6.88) years for the control group. There was no significant difference between the two groups regarding both sex and age. (Table 1)

**Table 1:** The intergroup comparisons of demographic data among both groups

Parameter		Study	Control	Test statistic	*p*-value
Gender n (%)	*Male*	8 (40.00%)	7 (35.00%)	**0.11**	**1**
	*Female*	12 (60.00%)	13 (65.00%)		
Age (years)	Mean ± SD	31.70 ± 7.81	31.85 ± 6.88	**0.06**	**0.949**
	*Median (IQR)*	32.50 (9.75)	31.50 (9.25)		

SD: Standard deviation. *Significant (*p* < 0.05)

### Clinical results

All the patients in both groups tolerated the operative procedure. Minute bleeding was quickly controlled, with variable degrees of pain during needle insertions and dextrose solution deposition. Those patients who demonstrated apparent muscular twitches during needle insertion were vulnerable to excessive pain.

The immediate postoperative phase showed variable degrees of transient lagophthalmos, abnormal or incomplete eyelid closure caused by the anesthetic effect contained in the injected solution among the test group patients on the temporal and zygomatic branches of the facial nerve, which was resolved entirely after three to four hours.

The patients in both groups demonstrated a course of postoperative pain, particularly upon mouth opening, throughout the few postoperative days, which was resolved after and seemed invariable among both groups. On the other hand, the patients in the test group complained of an altered occlusion for a few days after each dextrose solution injection. By the end of the study, all the patients expressed an apparent improvement in pain reduction as recorded by VAS, the MIO, and clicking sounds from the baseline records.

### Statistical results

By the end of the study, both groups showed a significant reduction in pain score values, (MIO) and clicking sounds from the baseline record.

**Fig. 2:**
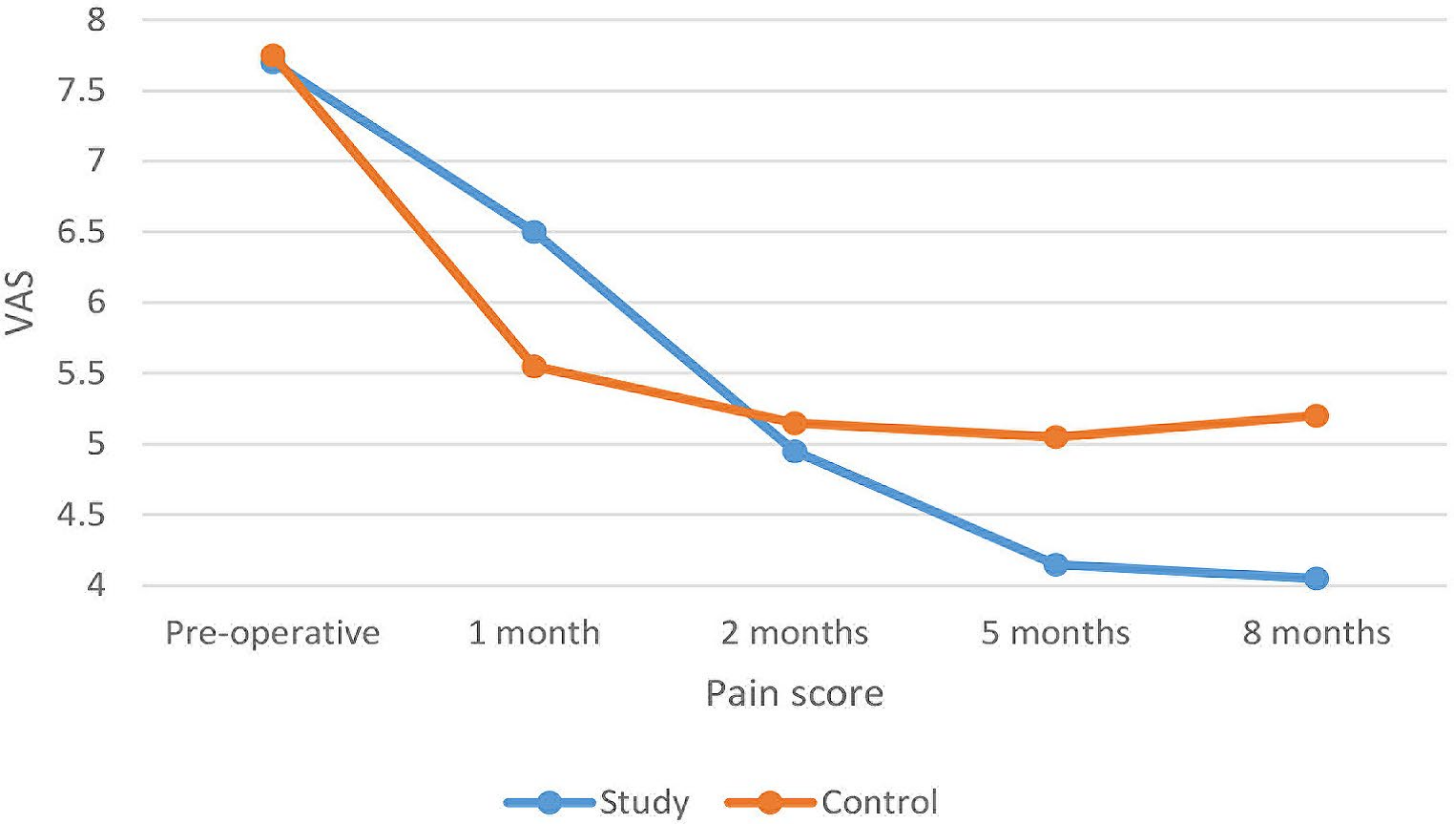
A line chart showing mean (VAS) values among both groups over different time intervals

The inter- and intragroup comparison results for the VAS pain score values showed that after one month, the pain scores measured in the study group were significantly higher than those of the control group (*p* = 0.009). The mean value of VAS pain score was recorded (6.50 ± 1.24) for the study group versus (5.55 ± 1.05) for the control group. There was no statistically significant difference between the VAS pain score values among both groups after two months. On the other hand, the pain score values of the control group after five and eight months were significantly higher than those of the study group (*p* < 0.05), recording (5.05 ± 1) and (5.2 ± 1) versus (4.15 ± 0.99) and (4.05 ± 0.76) respectively. (Table 2; Fig. 2)

**Table 2:** The inter and intragroup comparisons of the (VAS) pain score among both groups

Interval	Measurement	Study	Control	Test statistic	*p*-value
Pre-operative	*Mean ± SD*	7.70 ± 0.86^A^	7.75 ± 0.85^A^	**206.50**	**0.858**
	*Median (IQR)*	8.00 (1.00)^A^	8.00 (1.00)^A^		
1 month	*Mean ± SD*	6.50 ± 1.24^A^	5.55 ± 1.05^B^	**294.00**	0.009*
	*Median (IQR)*	7.00 (1.00)^A^	6.00 (1.00)^B^		
2 months	*Mean ± SD*	4.95 ± 1.15^B^	5.15 ± 0.93^B^	**181.00**	**0.600**
	*Median (IQR)*	5.00 (2.00)^B^	5.00 (1.25)^B^		
5 months	*Mean ± SD*	4.15 ± 0.99^B^	5.05 ± 1.00^B^	**291.00**	0.011*
	*Median (IQR)*	4.00 (2.00)^B^	5.00 (2.00)^B^		
8 months	*Mean ± SD*	4.05 ± 0.76^B^	5.20 ± 1.01^B^	**320.50**	< 0.001*
	*Median (IQR)*	4.00 (1.25)^B^	5.00 (1.25)^B^		
Test statistic		**68.89**	**64.49**		
*p*-value		< 0.001*	< 0.001*		

SD = Standard deviation, IQR = Interquartile range, Values with **different upper and lowercase superscript letters** within the **same horizontal row and vertical column** respectively are significantly different, *Significant (*p* < 0.05)

Within both groups, there was a significant difference in VAS pain values measured in different intervals (*p* < 0.05). For the study group, post hoc pairwise comparisons showed a significant reduction of the recorded VAS pain scores starting from 2 months until the end of the follow-up period, while for the control group, they showed the reduction of pain started after one month.

**Fig. 3:**
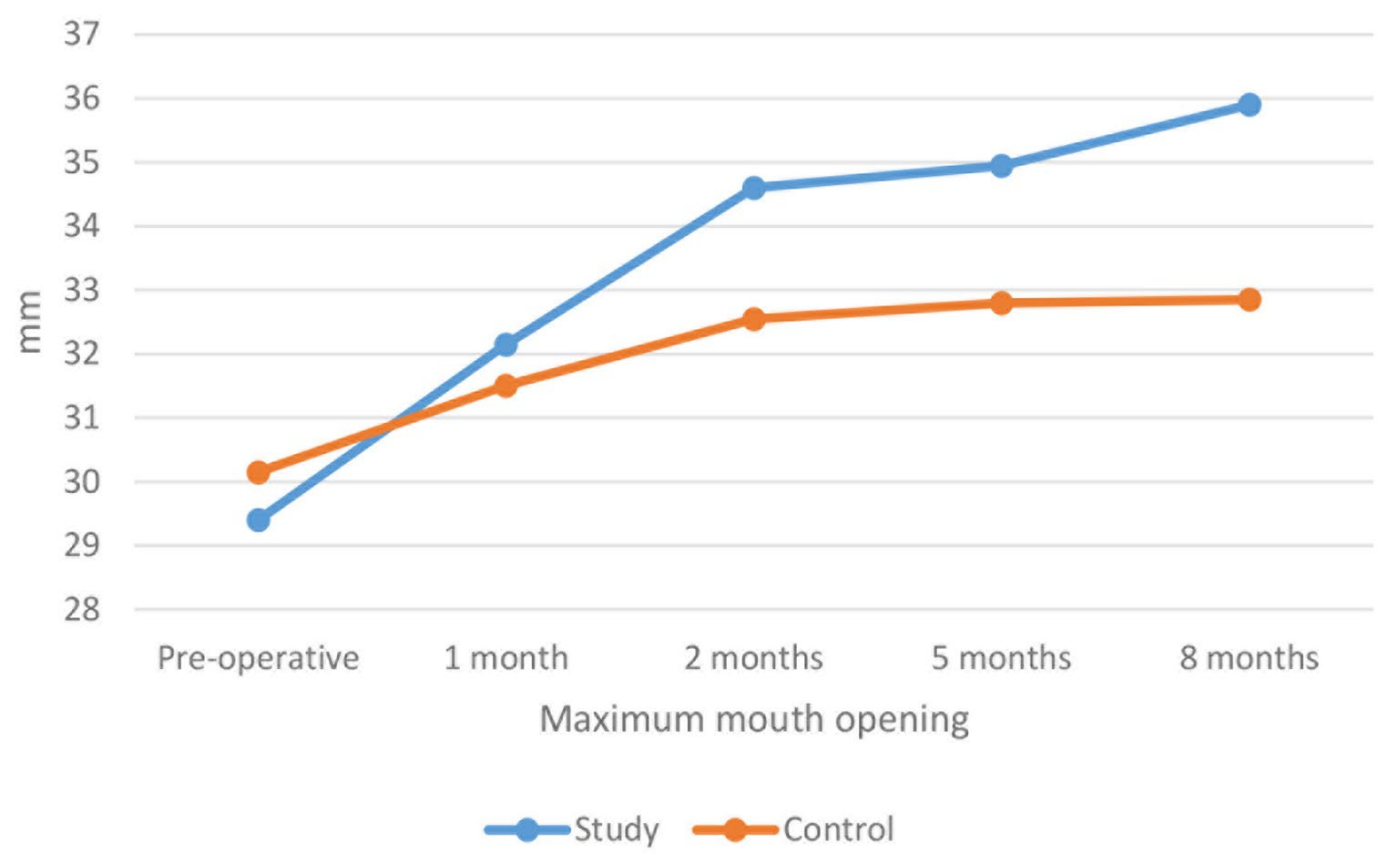
A line chart showing the mean (MIO) among both groups over the different time intervals

The results of the intragroup comparisons of the mean MIO calibration in millimeters demonstrated that there was no statistically significant difference among both groups in MIO at the time interval of one month (*p* > 0.05); (32.15 ± 1.18 mm) for the study group versus (31.50 ± 2.3 mm) for the control group. However, throughout the time intervals of two months, five months, and eight months, the study group demonstrated more significant MIO than that of the control group (*p* < 0.001), (34.60 ± 1.4 mm) for the study group versus MIO of (32.55 ± 2.09 mm) for the control group after two months, (34.95 ± 1.61 mm) MIO for the study group versus MIO of (32.80 ± 1.96 mm) for the control group after five months and MIO of (35.90 ± 1.48 mm) for the study group versus (32.85 ± 2.13 mm) MIO for the control group after eight months. (Table 3; Fig. 3)

**Table 3:** The inter and intragroup comparisons of the mean (MIO) in mm. Among both groups

Interval	Measurement	Study	Control	Test statistic	*p*-value
Pre-operative	*Mean ± SD*	29.40 ± 1.90^D^	30.15 ± 1.93^C^	**1.24**	**0.223**
	*Median (IQR)*	30.00 (3.00)^D^	30.00 (2.25)^C^		
1 month	*Mean ± SD*	32.15 ± 1.18^C^	31.50 ± 2.35^B^	**1.10**	**0.276**
	*Median (IQR)*	32.00 (2.00)^C^	30.50 (2.00)^B^		
2 months	*Mean ± SD*	34.60 ± 1.47^B^	32.55 ± 2.09^A^	**3.59**	< 0.001*
	*Median (IQR)*	34.50 (2.00)^B^	32.00 (2.25)^A^		
5 months	*Mean ± SD*	34.95 ± 1.61^AB^	32.80 ± 1.96^A^	**3.79**	< 0.001*
	*Median (IQR)*	35.00 (2.00)^AB^	33.00 (2.25)^A^		
8 months	*Mean ± SD*	35.90 ± 1.48^A^	32.85 ± 2.13^A^	**5.25**	< 0.001*
	*Median (IQR)*	36.00 (2.25)^A^	33.00 (2.75)^A^		
Test statistic		**90.21**	**38.87**		
*p*-value		< 0.001*	< 0.001*		

SD = Standard deviation, IQR = Interquartile range, Values with **different upper and lowercase superscript letters** within the **same horizontal row and vertical column**, respectively are significantly different, *Significant (*p* < 0.05)

Within both groups, there was a significant difference in values measured in different intervals (*p* < 0.001). For the study group, post hoc pairwise comparisons showed that the MIO values measured after eight months were significantly higher than those measured at other intervals except for five months (*p* < 0.001). In addition, MIO values measured after two and five months to be significantly higher than those measured pre-operatively and after one month (*p* < 0.001). Finally, the MIO values measured after one month to be significantly higher than the pre-operative value (*p* < 0.001).

For the control group, the MIO values values measured after two, five, and eight months were significantly higher than values measured pre-operatively and after one month (*p* < 0.001). In addition, the MIO values measured after one month were considerably higher than the pre-operative value (*p* < 0.001).

**Fig. 4:**
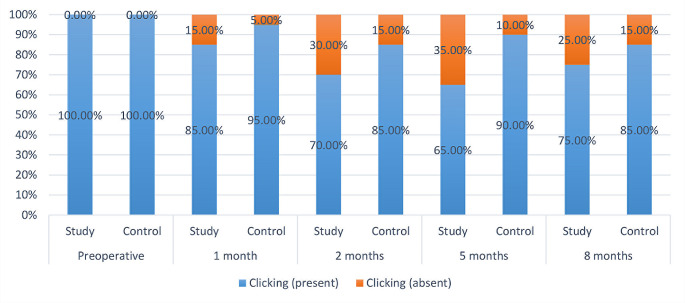
Stacked bar chart showing the presence of clicking among both groups at the different time intervals

The inter and intragroup comparisons for the presence or absence of joint clicking demonstrated that regardless of measurement interval, there was no significant difference between both groups regarding the presence of joint clicking (*p* > 0.05). (Table 4; Fig. 4)

**Table 4:** The inter and intragroup comparisons of the presence and absence of clicking

Interval	Clicking	n (%)		Test statistic	*p*-value
		Study	Control		
Pre-operative	*Presence*	20 (100.00%)^A^	20 (100.00%)^A^	**NA**	**NA**
	*Absence*	0 (0.00%)	0 (0.00%)		
1 month	*Presence*	17 (85.00%)^B^	19 (95.00%)^A^	**1.11**	**0.497**
	*Absence*	3 (15.00%)	1 (5.00%)		
2 months	*Presence*	14 (70.00%)^B^	17 (85.00%)^A^	**1.29**	**0.256**
	*Absence*	6 (30.00%)^B^	3 (15.00%)		
5 months	*Presence*	13 (65.00%)	18 (90.00%)^A^	**3.58**	**0.058**
	*Absence*	7 (35.00%)^B^	2 (10.00%)		
8 months	*Presence*	15 (75.00%)^B^	17 (85.00%)^A^	**0.62**	**0.429**
	*Absence*	5 (25.00%)	3 (15.00%)		
Test statistic		**14.67**	**7.56**		
*p*-value		0.005*	**0.109**		

NA: Not Applicable, Values with **different upper and lowercase superscript letters** within the **same horizontal row and vertical column** respectively are significantly different, *Significant (*p* < 0.05)

**Fig. 5:**
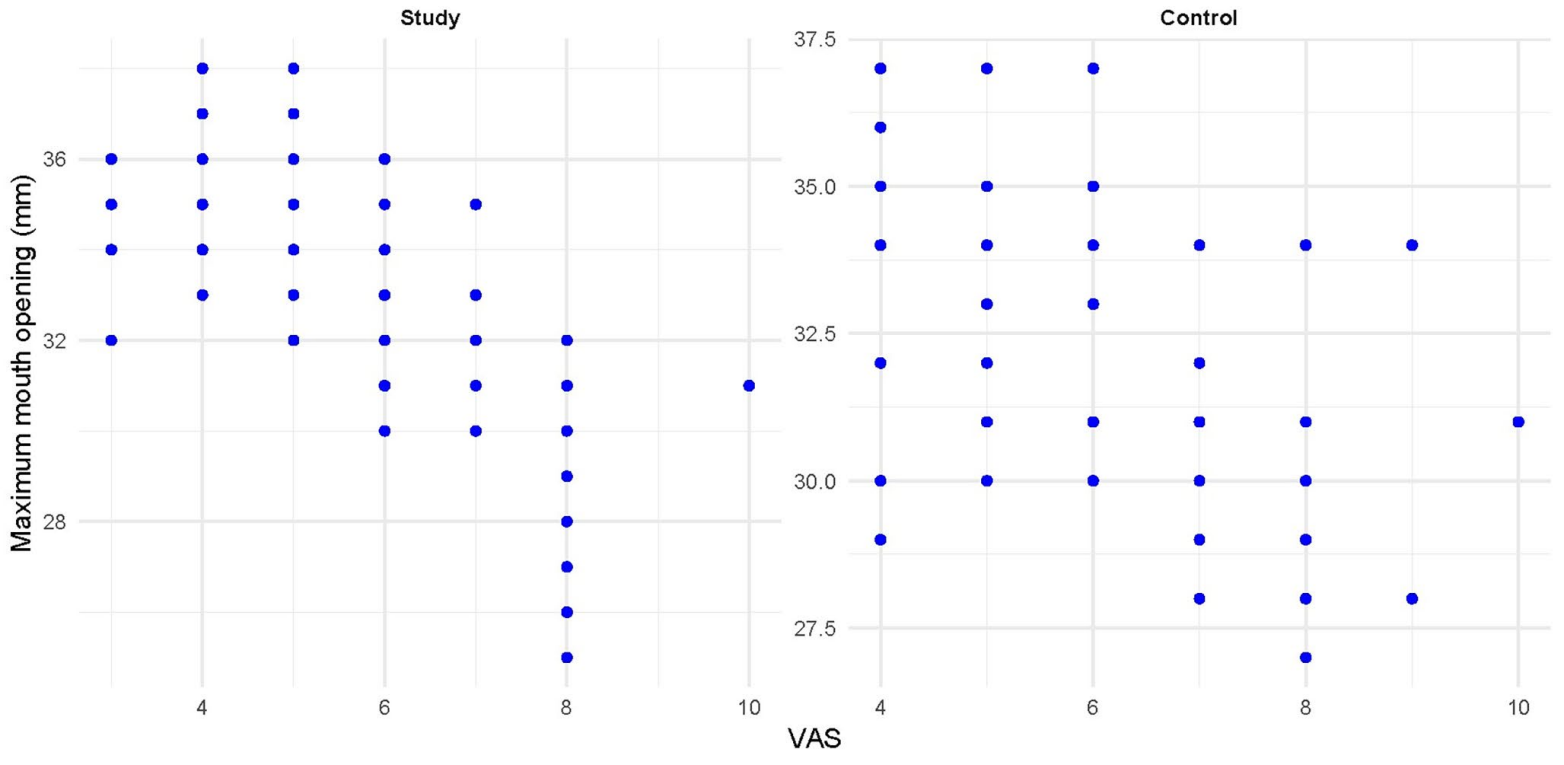
Scatter plot showing the correlation between (MIO) and mean (VAS) values within different groups

The correlations between the mean VAS pain values and increased MIO are presented in (Table 5; Fig. 5). For the study group, there was a strong negative correlation between both variables (rs>-0.5, *p* < 0.001), while for the control group, the correlation was weak (rs=-0.267, *p* = 0.007).

**Table 5:** The correlations between mean (VAS) pain values and mean (MIO)

Group	Correlation coefficient (95% CI)	Test statistic	*p*-value
Study	-0.626 (-0.732:-0.489)	270926.85	< 0.001*
Control	-0.267 (-0.440:-0.074)	211139.54	0.007*
Overall	-0.510 (-0.606:-0.400)	2013941.51	< 0.001*

CI = Confidence interval, *Significant (*p* < 0.05)

**Fig. 6:**
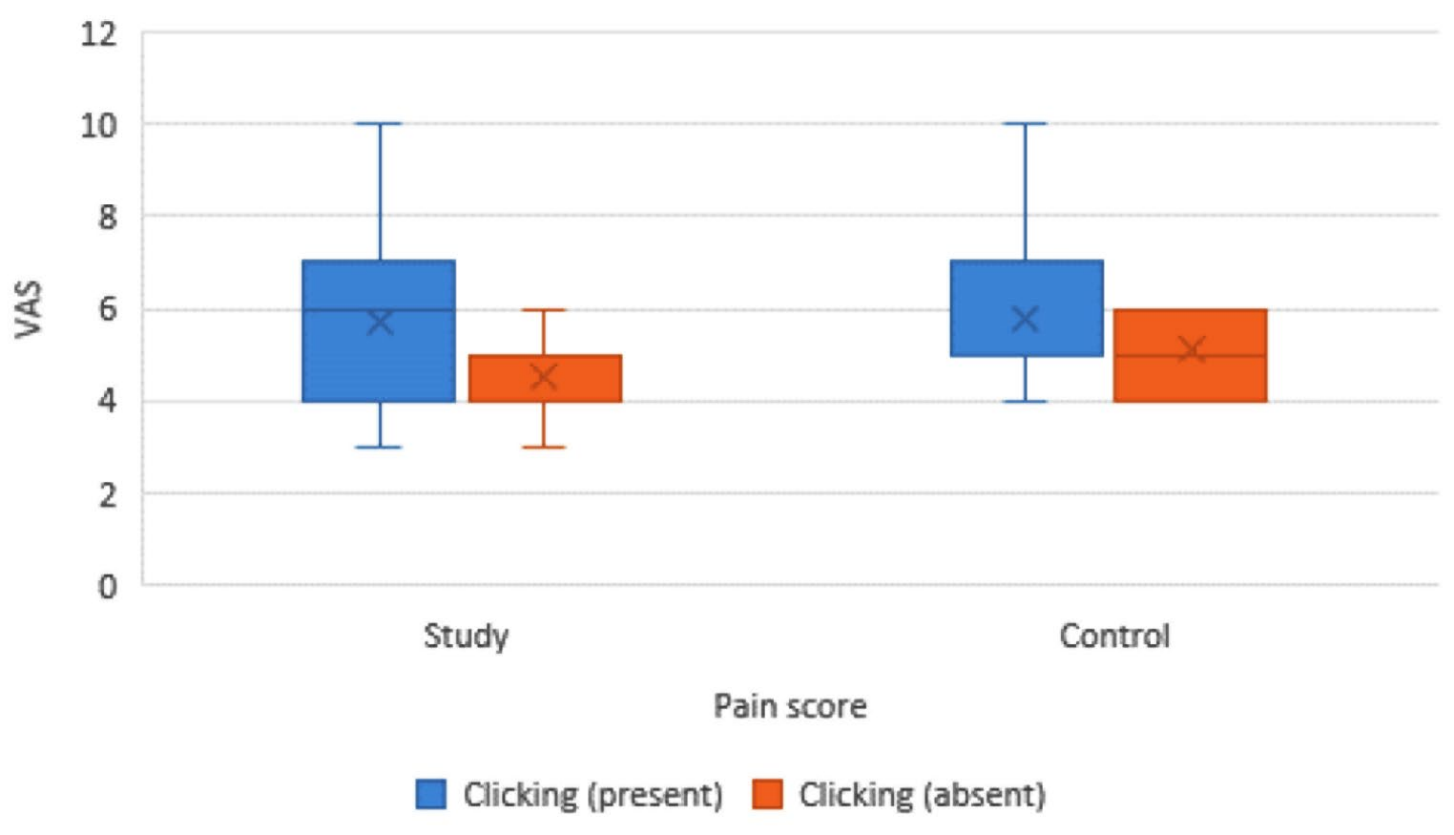
Box plot showing the association between the mean (VAS) values and clicking in both groups

The associations between VAS pain values and presence or absence of clicking are presented in (Table 6; Fig. 6). For the study group, the association was statistically significant among the cases diagnosed with persisting clicking and having higher pain scores (*p* < 0.05). In contrast, for the control group, the association was not statistically significant (*p* = 0.190).

**Table 6:** The associations between clicking and the mean (VAS) pain values among both groups

Group	Measurement	Clicking	No clicking	Test statistic	*p*-value
Study	*Mean ± SD*	5.72 ± 1.83	4.52 ± 0.87	**1144.00**	0.007*
	*Median (IQR)*	6.00 (3.00)	4.00 (1.00)		
Control	*Mean ± SD*	5.80 ± 1.42	5.11 ± 0.93	**516.00**	**0.190**
	*Median (IQR)*	5.00 (2.00)	5.00 (2.00)		
Overall	*Mean ± SD*	5.76 ± 1.62	4.70 ± 0.92	**3533.00**	< 0.001*
	*Median (IQR)*	6.00 (2.00)	5.00 (1.00)		

SD = Standard deviation, IQR = Interquartile range, *Significant (*p* < 0.05)

**Fig. 7:**
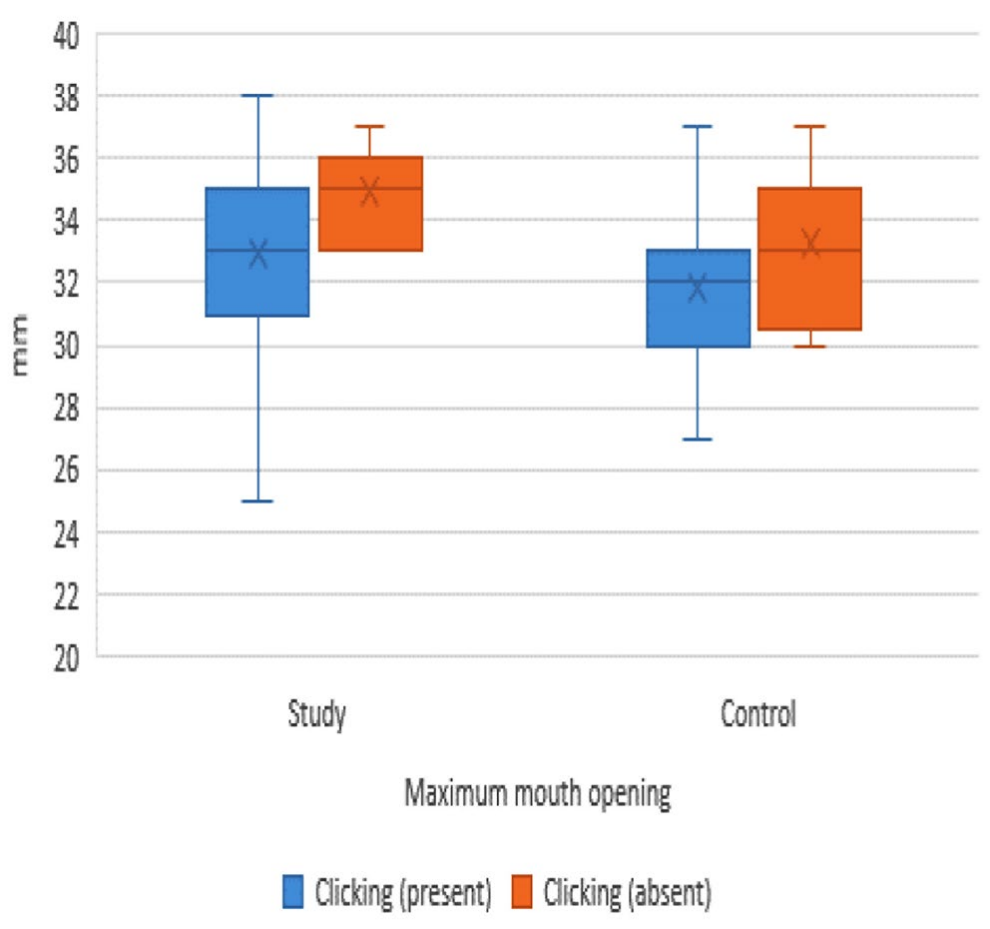
Box plot showing the association between the mean (MIO) and clicking in both groups

The associations between MIO and the presence or absence of clicking, as presented in (Table 7; Fig. 7), have significant implications. In the study group, the association was statistically significant, with cases diagnosed free from joint clicking having significantly higher (MIO) values (*p* < 0.05). In contrast, for the control group, the association was not statistically significant (*p* = 0.087). These findings could potentially influence future research and clinical practice in this area.

**Table 7:** The associations between clicking and mean (MIO)

Group	Measurement	Clicking	No clicking	Test statistic	*p*-value
Study	*Mean ± SD*	32.99 ± 2.93	34.95 ± 1.50	**2.96**	0.004*
	*Median (IQR)*	33.00 (4.00)	35.00 (3.00)		
Control	*Mean ± SD*	31.85 ± 2.26	33.22 ± 2.49	**1.73**	**0.087**
	*Median (IQR)*	32.00 (3.00)	33.00 (4.00)		
Overall	*Mean ± SD*	32.38 ± 2.65	34.43 ± 1.98	**4.05**	< 0.001*
	*Median (IQR)*	32.00 (4.00)	35.00 (3.00)		

SD = Standard deviation, IQR = Interquartile range, *Significant (*p* < 0.05)

## Discussion

This clinical trial is unique in its approach, focusing solely on the effect of the dextrose prolotherapy solution. Unlike previous studies, which often associate the solution with the needling impact, this trial investigates the solution’s effects independent of the needling process. The study compares the clinical effects of dry needling the anterior and posterior aspects of the mandibular condyle and the masseteric trigger point with the deposition of the Prolotherapy. Both methods utilize the same 25 gauge hypodermic needle and 3.8 cm length, and the same needling technique at the same time intervals. The dextrose prolotherapy demonstrated more significant, sustained, and correlated long-term alleviation of constitutional symptoms and increased maximal mouth opening.

Travell and Simons [12] originally described DDN therapy and utilized (22- gauge 1.5 – inch) hypodermic needles for TP obliteration without the deposition of an injectate. They believed the filiform acupuncture needles were too thin to disrupt the dense TPs. Hence, they preferred utilizing hypodermic needles as they provided optimal needling strength and tactile feedback. Hong [11] also implemented (27- gauge, 1.25-inch) hypodermic needles to compare the DDN effect versus Lidocaine injection into the TPs.

Although the DDN technique originally utilized hypodermic needles due to the thoughts that the solid filiform needles are easily deflected by the dense TPs contraction knots as they neither had proper strength nor provide acceptable tactile feedback, many dry needling practitioners believe that they better disrupt the TPs with less patient discomfort [13].

Fortunately, the use of filiform and hypodermic needles is documented for DDN practice. The use of the hypodermic needles here enabled the authors to utilize them in both dry and wet needling to identify the sole effect of the Dextrose Prolotherapy solution.

While selecting a needle for DDN, it should be long enough to penetrate the depth of the TPs. Concerning the needle diameter, Wang et al. [14] considered the needle diameter usually attributed to the personal preference and the experience of the operator. Larger diameter needles can penetrate the full thickness of the dense muscular knots better and provide a better tactile sense. While the needle with small diameters better guarantees the damage of the TPs when precisely placed. The authors here preferred the selection of 25-gauge needles as the 27-gauge needle utilized by Hong [10] carried out the risk of needle deflection, and the 22-gauge needle originally used by Travell and Simons [12] was too painful to be inserted into the temporomandibular joint.

The VAS pain score analysis demonstrated that the patients within both groups expressed noticeable improvement and pain relief from the baseline values. This could be attributed among the patients of the test group to the intraarticular deposition of the dextrose solution directly into the joint ligaments and the articular disc and into the masseteric TPs, which comes following Refai [11, 15], who considered that the dextrose injection precipitates both inflammatory and noninflammatory mechanisms that refer to both regenerative and growth-factor stimulation injection therapies, that promotes tissue repair and growth. On the other hand, the improvement of pain in the control group attributed to the ability of dry-needling to induce biochemical and mechanical as well as neurovascular changes that elicit a reduction in pain intensity and disability, as per Butts et al. [16].

Although the patients within the control group exhibited less pain than those of the test group during the first month of treatment, and there was an invariable improvement in the second month, the pain reduction among the test group patients exceeded those of the control group after five and eight months. This follows what is believed by Rabago et al. [17], that the beneficial prolotherapy effect is a long-term one that requires multiple injections delivered every 2 to 4 weeks over a treatment course of several months.

Dasukil et al. [18] considered prolotherapy an alternative to long-term narcotic administration or TMJ surgery by relieving persistent and refractory joint symptoms as a result of the repair of the joint ligaments and capsules. They attributed the marked improvement in mouth opening after three months of treatment to the alleviation of pain and the reduction in the surface friction and the viscosity of the synovial fluid. This coincides with the current study’s findings, as although both groups demonstrated invariable increases MIO during the first month, the patients treated with the deposition of the dextrose solution exhibited a marked gradual increase in mouth opening beginning from the second month. We attribute the increase in MIO after both dextrose infusion and DDN to the physiological alteration induced by either treatment rather than the pain reduction, as the correlation between pain reduction and increased MIO was not evident in the test group and very weak in the control one.

The gradual increase of the MIO was evident for both treatment maneuvers, as the authors reported a linear increase in the mouth opening throughout the follow-up period among the test group. However, it was more apparent for the patients who received the dextrose solution deposition,

Hakala and Lederman [19] evaluated the effect of Dextrose prolotherapy for temporomandibular joint disorders (TMDs) and reported a (78%) improvement percentage in clicking. Priyadarshini et al. [20] compared the impact of prolotherapy versus occlusal splints in reducing pain and TMJ clicking in patients with internal derangement. They preferred the deposition of Dextrose Prolotherapy solution as they documented an improvement in clicking in 90% of patients. Dib-Zakkour et al. [21] evaluated DDN ‘s effectiveness in treating Myogenous TMDs. They reported better muscular harmony due to the depletion of muscle hyperactivity after needling its trigger points, which reduced pain and clicking.

The results of this study match these findings. The authors here detected a marked disappearance of TMJ sounds among the test and control groups with no statistically significant difference between either group at any time interval. On the other hand, the reduction of TMJ clicking was correlated to both the pain reduction and the mouth opening increase in the test group, while in contrast, it was not correlated to either pain reduction or mouth opening increase in the control group. This reflects an image that the more generalized multifactorial mechanism of action of the dextrose prolotherapy would precipitate an environment capable of regenerating the complex, diversified composition of the TMJ in harmony. This matches what believed by Refai et al. [15] that although it is not fully understood how the deposition of an inflammatory level of hypertonic glucose would raise various growth factor levels and cause adequate cell wall lysis, enough to attract fibroblasts and begin an effective regenerative process that ends with tightening of loose ligaments, toning down stiff muscles and enhancing the joint mechanics.

Steilen et al. [22] considered that prolotherapy represents a prolonged regenerative injection technique that induces the tissues to regenerate for months after the deposition of the hypertonic solution because it is based on a complex mechanism of action that overlap the three stages of the regenerative healing of inflammation, proliferation, and remodeling. This explains the presence of a lag of time before the gradual alleviation of the symptoms in the current study. In contrast to the faster alleviation of pain encountered among the patients of the control group, as explained by Alvarez and Rockwell [23] DDN induces a mechanical disruption of the TPs as a result of the depolarization of the nerve membranes provoked by the intercellular Potassium release that interrupts the central feedback mechanism.

Considering that all the patients within both groups were subjected to needle insertion in the same manner, it seemed that the deposition of the prolotherapy solution, although deferring the fast-relieving dry needling effect among the patients of the test group, provoked a steady, sustained, and significant long-term improvement in pain reduction and mouth opening increase.

Although the limited sample size of this clinical trial would affect the reliability of the final results, the study represents the first trial to isolate the sole effect of intraarticular and TPs injection of Prolotherapy dextrose solution, apart from the unavoidable associated dry needling effect.

In conclusion, dextrose prolotherapy injection and dry needling are simple, safe, and beneficial treatment modalities for TMJ anterior disc displacement with reduction. However, the deposition of the Dextrose Prolotherapy solution, preceded by dry needling, demonstrated more significant, sustained, and correlated long-term alleviation of constitutional symptoms and increased maximal mouth opening, rejecting the null hypothesis that there is no difference between both groups.

## Electronic supplementary material

Below is the link to the electronic supplementary material.

Supplementary Material 1

Supplementary Material 2

## Data Availability

The data that support the findings of this study are available from the corresponding author upon request.

## Abbreviations

TMJ: Temporomandibular joint
DDN: Deep Dry Needling
MIO: Maximal interincisal opening
VAS: Visual Analogue Scale
MRI: Magnetic resonance imaging
TPs: Trigger points
TMDs: Temporomandibular joint disorders
