# Supplementary material for: Evaluation of the effect of dextrose prolotherapy versus deep dry needling therapy for the treatment of temporomandibular joint anterior disc displacement with reduction: (a randomized controlled trial)
Source: Clin Oral Investig. 2024 Aug 8;28(9):475. doi: 10.1007/s00784-024-05830-z (PMC11310281; doi:10.1007/s00784-024-05830-z)
Supplement: Supplementary file 1 — Supplementary Material 1 [file 784_2024_5830_MOESM1_ESM.docx]

**Allocation**

Allocated to study group (n= 20)

- Received allocated intervention (n= 20)
- Did not receive allocated intervention (n= 20)

Allocated to control group (n= 20)

- Received allocated intervention (n= 20)
- Did not receive allocated intervention (n= 20)

Lost to follow-up (n= 0)

Lost to follow-up (give reasons) (n= 0)

Analyzed (n= 20)

- Excluded from ranalysis (n= 0)

Analyzed (n= 20)

- Excluded from analysis (n= 0)

**Analysis**

**Follow-Up**

Randomized (n= 40)

Excluded (n= 12)

- Not meeting inclusion criteria (n= 12)

Assessed for eligibility (n= 52)

**Enrollment**
